# Editorial: Biological and functional restoration of mechano- and electro conductive tissues and organs: A regenerative approach

**DOI:** 10.3389/fbioe.2022.1070888

**Published:** 2022-11-03

**Authors:** Yun Qian, Xuqing Liu, Gonzalo Rosso, Peisheng Xu

**Affiliations:** ^1^ Department of Orthopedics, Shanghai Sixth People’s Hospital Affiliated to Shanghai Jiao Tong University School of Medicine, Shanghai, China; ^2^ Department of Materials, University of Manchester, Manchester, United Kingdom; ^3^ Max Planck Institute for the Science of Light and Max-Planck-Zentrum für Physik und Medizin, Erlangen, Germany; ^4^ Department of Drug Discovery and Biomedical Sciences, University of South Carolina, Columbia, SC, United States

**Keywords:** pharmacological therapy, mechanical and electrical stimuli, conductive materials, tissue engineering, regenerative medicine

The primary mission of tissue engineering and regenerative medicine (TERM) is to mimic the architectural and functional nature of impaired tissues (Berthiaume et al., 2011). TERM has made up significantly for the shortage of organs and tissues after severe trauma or terminal illness. Nevertheless, the complete functional and biological recovery of tissues and organs is still limited due to failure to restore *in vitro* and *in vivo* biomimetic scenarios based on tissue engineering. Among various regenerative cues, including chemical, biological, optical, magnetic and mechanical factors, the implementation of advanced pharmacological approaches, electrical and mechanical stimuli have long been underestimated in regard to their potential for the development and improvement of bioengineered and biological tissues, such as the bone, cartilage, muscle, heart, and nerve.

Mechanical and electrical activities play a crucial role in a series of physiological phenomena in the living body and are important for the functionality of mechano- and electro-active tissues, such as bone, cartilage, muscle, heart, brain, spinal cord, and peripheral nerve. Therefore, it is vital to focus on the application of conductive scaffolds and their regulation on endogenous electrical activities in the process of tissue regeneration, with or without exogenous mechanical and electrical stimuli of different paradigms (e.g., intensity, frequency, and wave type). Positive outcomes have been reported in previous literature, but it is poorly understood how electrical phenomena affect cell physiological function-behavior, metabolism, signaling transduction, and gene expression, or how the combination of engineered conductive scaffolds with the specific delivery of therapeutic drugs boosts the regenerative capacity of tissues. For instance, the inter-cellular communication between neurons or glial cells influenced by electrically conductive scaffolds is not well elucidated in nerve tissue engineering. Some preliminary findings were obtained from *in vitro* studies. Long-term evaluation on the reparative potential of mechanically and electrically conductive biomaterials is the key to identifying a translational approach to advance the field of mechano- and electro-active tissue regeneration therapies.

In this Research Topic, we have covered the latest advances in the modulation of electrophysiological activities of cells, tissues, and organs by conductive biomaterials and their regenerative signaling mechanisms. This has ultimately led to a comprehensive display of papers (10 articles from China, the United Kingdom, Germany, and Australia).

Some papers focused on physiological and metabolic response of excitable and non-excitable cells and tissues on electrically active substrates under mechanical and electrical stimuli in normal and tissue injury environments. Liu et al. stressed the involvement of osteoclasts and osteoblasts in the activation of various mechanical transduction pathways and discussed changes in the differentiation, formation, and functional mechanisms under multiple forms of mechanical stress to bone tissues. Mei et al. designed a mesoporous bioactive glass biomimetic scaffold to enhance cellular adhesion and to improve osteogenic/cementogenic differentiation in human periodontal ligament cells.

Wang F et al. proposed a conductive adhesive and anti-bacterial Zwitterionic hydrogel dressing to repair full-thickness skin wounds. In their study, modulation of electrical paradigms facilitated intercellular communication and transcriptional signaling, mechanical stimulation for angiogenesis and skin development and function supported by conductive scaffolds. Tian Y et al. claimed pro-healing effects of mechanically conductive tissue engineering strategy with bardoxolone methyl on nucleus pulposus cells and tissues by inhibiting extracellular matrix (ECM) catabolism and promoting ECM anabolism. In addition to traditional electrical stimuli, *in vitro* and *in vivo* evaluation of wound healing and tissue regeneration technologies of combined electrical stimulation and smart materials also yielded some promising results, including novel electroactive scaffold design and user-friendly application (Nan et al.; Xiong et al.).

Jiang Y et al. reported the Netrin-1 modified adipose-derived stem cells and the combined application of such *in vitro* and *in vivo* tissue engineering strategies to accelerate or improve the development and function of bioengineered endovascular tissues. In addition, there are some research papers concerning novel biomaterial design, fabrication and application for mechanobiology/electrobiology and interfacial characterization of these biomedical materials in bone and nerve regeneration (Liu et al.; Yan et al.; Gao et al.).

The improvement in tissue regeneration, either in functionality or morphology, largely depends on the increasing knowledge of the properties of mechano- and electro conductive tissues and organs. The purpose of physical stimulation is generally to simulate the nature of the living tissue and organ itself. For instance, microenvironmental remodeling in nerve repair is essential and influenced by the spatial and temporal changes after nerve injury. This also greatly affects the scaffold design and physiochemical factors of the nano- and micro-scale biomaterials (Qian et al., 2021). The recent progress from this Research Topic provides novel insights into the regenerative approach from a physical or mechanical perspective for various cells and tissues. Some of the exciting techniques and advanced concepts may be valuable for inspiring more applications for potential clinical translation in the near future.
